# Role of NKT Cells during Viral Infection and the Development of NKT Cell-Based Nanovaccines

**DOI:** 10.3390/vaccines9090949

**Published:** 2021-08-26

**Authors:** Masood Alam Khan, Arif Khan

**Affiliations:** Department of Basic Health Sciences, College of Applied Medical Sciences, Qassim University, Buraydah 51452, Saudi Arabia; 4140@qu.edu.sa

**Keywords:** NKT cells, vaccine, lipid ligands, immunoadjuvant, nanoparticles

## Abstract

Natural killer T (NKT) cells, a small population of T cells, are capable of influencing a wide range of the immune cells, including T cells, B cells, dendritic cells and macrophages. In the present review, the antiviral role of the NKT cells and the strategies of viruses to evade the functioning of NKT cell have been illustrated. The nanoparticle-based formulations have superior immunoadjuvant potential by facilitating the efficient antigen processing and presentation that favorably elicits the antigen-specific immune response. Finally, the immunoadjuvant potential of the NKT cell ligand was explored in the development of antiviral vaccines. The use of an NKT cell-activating nanoparticle-based vaccine delivery system was supported in order to avoid the NKT cell anergy. The results from the animal and preclinical studies demonstrated that nanoparticle-incorporated NKT cell ligands may have potential implications as an immunoadjuvant in the formulation of an effective antiviral vaccine that is capable of eliciting the antigen-specific activation of the cell-mediated and humoral immune responses.

## 1. Introduction

**Figure 1:**
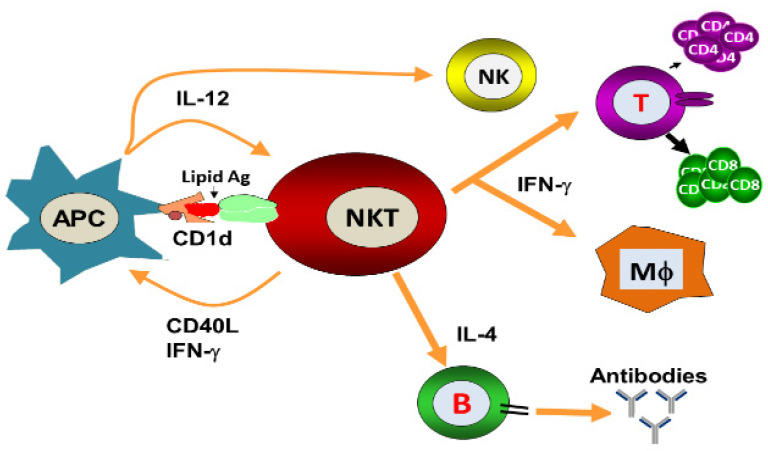
Activation of NKT cells results in the secretion of Th1 and Th2 cytokines. Lipid antigens presented by CD1d activate NKT cells, resulting in the secretion of IFN-γ and IL-4. IFN-γ activates the T cells and macrophages that are important players in cell-mediated immunity, whereas IL-4 acts on B cells and contributes to humoral immunity.

**Figure 2:**
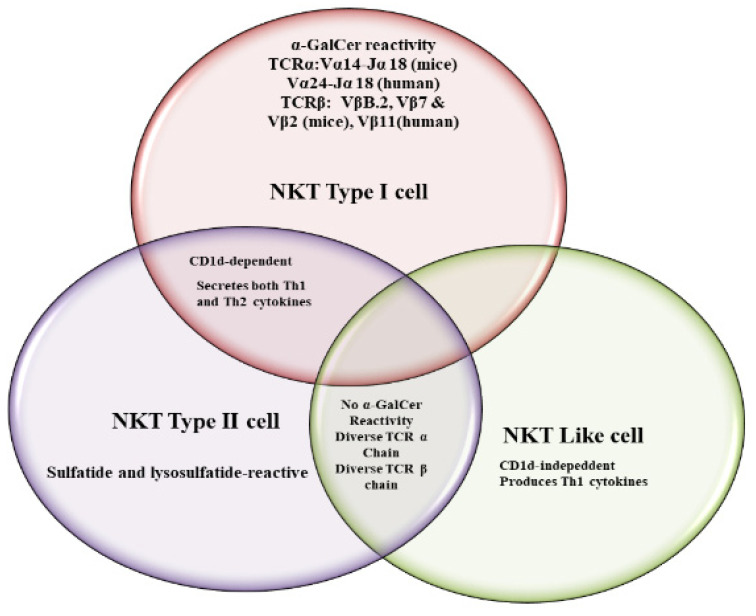
Similarities and differences between different types of NKT cells.

Natural Killer T (NKT) cells belong to a subset of T cells that can influence the status of the innate and adaptive immune systems because they secrete huge amounts of Th1 and Th2 cytokines (Figure 1) [1]. Earlier, the NKT cells were characterized by the NK and T cell properties as they express the natural killer (NK) cell lineage markers (NK1.1 or DX5 in mice and CD161 in human) and αβ T-cell receptor (TCR) [1,2]. NKT cells are more appropriately defined as “CD1d-restricted and TCR-αβ positive T cells”. In mice, the NKT cells constitute about 0.2–2.0% of lymphocytes in the blood, spleen, bone marrow and thymus, and about 15–35% of total lymphocytes in the liver. On the other hand, the levels of NKT cells are lower in humans, comprising about 0.04–1.3% of circulating lymphocytes in the blood, spleen and bone marrow. They make up about 0.001–0.01% of lymphocytes in the thymus and about 1% in the liver [3]. The greater part of the NKT cells, called canonical or invariant NKT cells (iNKT cells) or type I NKT cells have a specific TCR α-chain rearrangement (Vα14-Jα18 in mice; Vα24-Jα18 in humans), associated with limited diverse Vβ chains (Figure 2). Type II NKT cells, also called non-classical NKT cells, are more diverse in TCR α-chain (but some Vα3.2-Jα9, Vα8 in mice) and TCR-β chains (but some Vβ8.2 in mice) (Figure 2).

NKT cells have the ability to recognize lipid or glycolipid antigens that are presented by the non-classical Major histocompatibility complex (MHC) I-like CD1 molecule [1]. The professional antigen-presenting cells (APCs), including macrophages, B cells and dendritic cells express the CD1 molecules. The CD1 family consists of two groups: group 1 CD1 includes CD1a, CD1b, and CD1c, whereas CD1d is the only member of CD1 group 2. There are two CD1d molecules (CD1d1 and CD1d2) in mice. CD1d molecules bind to their specific ligands that activate the NKT cells [1]. Contrary to type I and II NKT cells, the NKT-like cells are CD3^+^ CD56^+^ and are independent of CD1d (Figure 2) [4]. The NKT-like cells express the diverse TCR-α and TCR-β chains. They are nonreactive to α-Galcer and secrete Th1 cytokines. They are not detectable in newborns, whereas their numbers are increased in elderly individuals [4]. In a recent report, Terrazzano G. et al. defined CD3^+^ CD56^+^ T cells as T_R3-56_ cells that play a regulatory role by controlling the effector function of CD8^+^ T cells. The frequency of T_R3-56_ cells was found to be reduced in type 1 diabetes [5].

Besides type I and type II NKT cells, other CD1d-restricted cells also express a semi-invariant Vα10-Jα50 TCR and are CD1d/α-GalCer tetramer-positive cells that have been demonstrated in *J*α*18*^−/−^ mice [6]. Mucosal-associated invariant T cells (MAIT Cells), a subset of T cells restricted to the MHC class I-related molecule (MR1-restricted T cells), are characterized by the expression of a semi-invariant TCR composed of a canonical TCRα chain (Vα19-Jα33 in mice and Vα7.2-Jα33 in humans) associated with a restricted set of Vβ segments [7]. Several subsets of the human and mouse MAIT cells have recently been identified using diverse αβ TCR. The MAIT cells can respond to TCR signals or to various activating cytokines, including IL-12, IL-1β, IL-18, and IL-23 [8,9,10]. Upon activation, MAIT cells produce huge amounts of Th1- and Th17-related cytokines, such as IFN-γ, TNF-α, IL-17A, and IL-22 [11]. Additionally, MAIT cells have the ability to kill bacteria-infected cells [12,13].

## 2. NKT Cell Ligands

NKT cells are activated by the specific exogenous or endogenous ligands presented by CD1d molecules. The most common exogenous CD1d ligand, α-Galactosylceramide (α-GalCer), was originally extracted from a marine sponge *Agelas mauritians* [14]. Later on, α-GalCer was also found to be secreted by the two human gut commensals, including *Bacteroides vulgatus* and *Prevotella Capri* [15,16]. Glycosphingolipids, from a lipopolysaccharide (LPS)-free bacteria *Sphingomonas paucimobilis*, activate the NKT cells in a CD1d-dependent manner [17]. Some microbial components such as lipophosphoglycan from *Leishmania donovani* andasperamide B from *Aspergillus fumigatus* can stimulate type I NKT cells, whereas sulfatide, lysophosphatidylcholine, lyso-GL-1, phosphatidylglycerol (PG), di-phosphatidylglycerol (DPG), phosphatidylinositol (PI) from *Corynebacterium glutamicum,* PG and DPG from *Listeria monocytogenes* and DPG from *Mycobacterium tuberculosis* can activate type II NKT cells [18,19,20]. Upon activation with specific ligands, NKT cells secrete a copious amount of Th1 (IFN-γ, IL-2 and GM-CSF) and Th2 (IL-4, IL-10 and IL-13) cytokines that act on the cells of the innate and adaptive immunity [21,22].

NKT cells play a very important role against a wide range of pathogens, including viruses, protozoans, bacteria and fungi [23]. The NKT cell ligands have been suggested to be effective immunoadjuvants in the formulation of synthetic vaccines [24]. Taking their immune-stimulatory role into consideration, NKT cell ligands may be very useful in the preparation of vaccine formulation and immunotherapeutics in order to prevent infectious diseases. Since NKT cells have a role in the regulation of the innate and adaptive immune responses, the pathogens try to evade the functioning of NKT cells in order to establish the infection. In the present review, we describe the antiviral roles of NKT cells and also discuss the implications of NKT cell-based nanoparticle vaccines to protect against viral infections.

## 3. Role of iNKT Cells against Viral Infections

NKT cells have antiviral potential against hepatitis B virus (HBV), respiratory syncytial virus (RSV), encephalomyocarditis virus (EMCV), Herpes simplex virus-1 (HSV-1), coxsackievirus B (CVB), lymphocytic choriomeningitis virus (LCMV), influenza A virus (IAV) and murine cytomegalovirus (MCMV) [25,26,27,28,29,30]. They constitute an important arm of the innate immune response against viruses and can also regulate the adaptive immune responses by modulating the antigen-presenting cells (APCs). NKT cells exert the direct cytolytic effects and restrict the replication of viruses. Moreover, they can indirectly induce an antiviral state through the secretion of important cytokines. α-GalCer-activated NKT cells reduced the replication of murine cytomegalovirus [30]. Likewise, α-GalCer administration protected the mice against viral encephalomyocarditis [27]. Wu et al. showed that α-GalCer protected the mice against coxsackievirus B3 (CVB3)-induced myocarditis [31]. Johnson et al. demonstrated that NKT cell activation has been shown to induce the expansion of Cytotoxic T lymphocytes (CTLs) response and enhance the immune response against RSV [32]. The activation of the NKT cell resulted in the reduction of the viral load in the pancreas of LCMV-infected mice and this effect was mediated through the secretion of type I interferon [28]. Whereas, α-GalCer-induced activation of NKT cells against HBV was shown to be mediated by IFN-α/β and IFN-γ secretion [25]. However, the antiviral effect of NKT cells against the IAV is mediated through the activation of the innate immune responses [29]. Type I NKT cells exhibited a critical role against IAV infection by increasing IAV-specific CD8^+^ T cell response and viral clearance [33]. Singh et al. showed that cytokines produced by iNKT cells were associated with non-progressive HIV-I infection and patients had a lower viral load in their plasma [34]. There have been many studies that support the role of NKT cells against viral infections in humans. For example, some patients with mutated adaptor protein SAP (signaling lymphocyte activation molecule-associated protein) were found to be deficient in invariant iNKT cells [35]. These patients have been found to be susceptible to EBV infection. In an another study, patients mutated in the X-linked inhibitor of the apoptosis protein (XIAP) showed the selective reduction in iNKT cell numbers without affecting B cells, T cells and NK cells [36]. The Wiskott−Aldrich syndrome (WAS), a primary immune deficiency disease in humans, has been shown to be associated with an iNKT cell deficiency that impaired the functioning of the innate and adaptive immune systems and therefore predisposed the patients to viral infections [37]. The NKT cells play a very important role in linking the innate and adaptive immunity, their deficiency causes severe immunodeficiency in those persons for whom vaccination with attenuated pathogens is a serious challenge. This was evident from a report that a girl with impaired iNKT cell number and function developed severe respiratory distress after vaccination with the attenuated varicella-zoster virus (VZV) [38].

NKT cells have been actively involved in immune responses against HSV-1 and HSV-2 as NKT cell-deficient mice showed severe HSV-1 infection and impaired viral clearance [39]. The immune evasion strategy of HSV includes the inhibition of NKT cell recognition by curbing the CD1d recycling from the late endosomal compartments to the cell surface [40]. The protective role of NKT cells against RSV was evident from the fact that Cd1d^−/−^ mice infected with RSV showed a poor ability to clear the viral load [39]. MCMV is considered a study model for human cytomegalovirus (HCMV). The iNKT cell activation by MCMV cells requires the involvement of IL-12 and type I interferon, but was independent of CD1d [41]. In an another study, a role of iNKT cells and CD1d has been suggested to counter the MCMV-induced suppression of hematopoiesis in mice [42]. The CD1d-dependent activation of NKT cells plays an important role in resisting EMCV infection because Cd1d^−/−^ mice demonstrated more brutal paralysis due to an acute cytopathic effect of EMCV on neuronal cells [27]. The iNKT cells also played an important role in protecting against IAV because iNKT cell-deficient mice showed more severe bronchopneumonia. The adoptive transfer of iNKT cells before the infection reversed the effect of IAV-induced bronchopneumonia [43]. 

The NKT cells are found in the highest numbers in the livers of mice and therefore are supposed to play a very important role in understanding the pathology of liver diseases [44]. The activation of NKT cells had a protective role against HBV infection [25,45]. The absence of NKT cells or CD1d in mice resulted in diminished HBV-specific T and B cell responses with delayed viral clearance [46]. Toll-like receptor (TLR) ligands have been shown to activate iNKT cells [47]. Viruses altered the antigen presentation by CD1d through the activation of pattern recognition receptors, such as TLRs. The endosomal TLRs (TLR 3, 7, 8, and 9) are involved in detecting viruses by recognizing the nucleic acid structures [48]. TLR-mediated recognition of viruses may lead to altered CD1d-mediated antigen presentation, thereby affecting the activation of iNKT cells. The role of NKT cells has been extensively reported against viral infections in pigs. The percentage of iNKT cells were increased in the blood, lymph node and broncho-alveolar lavage of pigs upon IAV infection [49]. Renukaradhya et al. demonstrated the role of iNKT cells in the regulation of airway hyper-reactivity [50]. The intranasal administration of NKT cell ligand ameliorated H1N1 IAV infection in piglets [51], while the adjuvant effect of α-GalCer potentiated the immune response of the inactivated HIN1 influenza virus in pigs [52].

## 4. Evasion of the NKT Cell Functioning by Viruses

**Figure 3:**
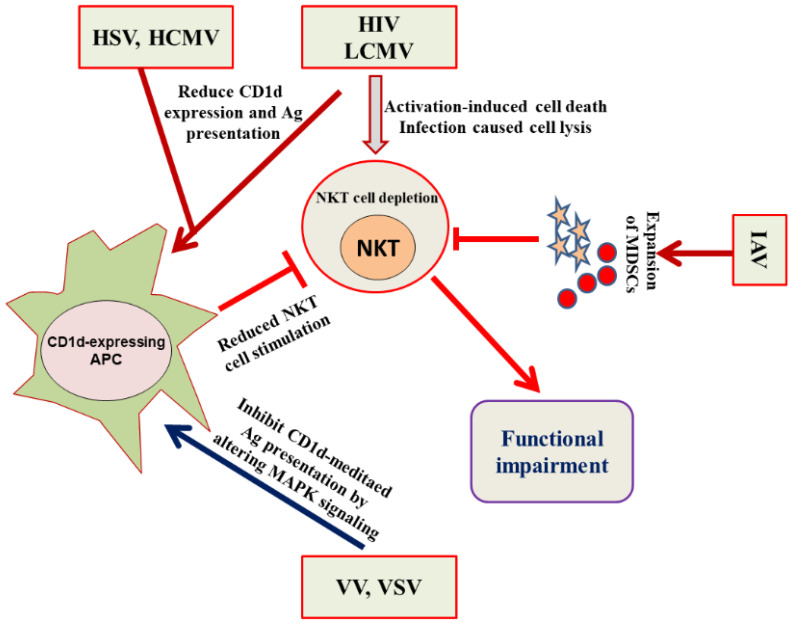
HIV, LCMV, HSV, HCMV, VV, VSV and IAV adopt different strategies to evade the NKT cell-mediated immune responses. HSV, HCMV, HIV and LCMV reduce the expression of CD1d on APCs. HIV and LCMV induce the activation-induced cell death in NKT cells. IAV induces the expansion of MDSCs that cause the impairment of NKT cell functioning. VV and VSV impede the NKT cell stimulation by inhibiting the CD1d-mediated antigen presentation.

Microbial pathogens can cause recurrent or persistent infections by avoiding the onslaught of the normal host immunity. Viruses adopt different strategies to evade both the innate and adaptive arms of the immune system (Figure 3). For example, HIV weakens the immune system by depleting the numbers of CD4^+^ T cells [53]. Some viruses, such as the herpes virus and Epstein−Barr virus (EBV), have the ability to enter into a latent state. Although the viruses do not replicate in the latent state, they cannot, however, be eliminated and can reactivate themselves to a fully virulent form. Invariant NKT (iNKT) cells have shown an antiviral immunity upon stimulation with α-GalCer against HIV, MCMV, RSV, HBV and influenza virus infections [25,26,27,28,29,30,54]. There has been a reported reduction in the iNKT cell numbers in HIV-1^+^ patients, particularly a depletion in the CD4^+^ iNKT cell subset [55]. Moreover, HIV also causes the functional impairment of iNKT cells as the CD4^+^ and CD4^−^ iNKT cells secrete a lower amount of IFN-γ, TNF-α, and IL-4 in response to α-GalCer/IL-2/PMA stimulation [56]. Viruses have adopted many tactics to avoid the assault by the immune system. For example, HIV-1 reduces the expression of CD1d molecules by increasing its internalization and retains them in the trans-Golgi network. The downregulation in the cell surface CD1d is caused by the interaction of CD1d intra-cytoplasmic tyrosine with HIV-1 Nef protein [57,58]. The CD4^+^ NKT cells showed greater susceptibility, as compared to conventional CD4^+^ T cells, to HIV-1 infection owing to the elevated level of CCR5 coreceptor expression on iNKT cells [59]. Fernandez et al. demonstrated an early NKT cell depletion in HIV-infected individuals [60]. Besides, iNKT cells showed their functional impairment as they produced lower levels of Th1 and Th2 cytokines in response to α-GalCer [61]. The functional status of NKT cells is significantly preserved in the long term nonprogressors (LTNPs) as compared to the progressors [34].

Cell signaling pathways play an important role in modulating the CD1d-mediated antigen presentation to NKT cells by viruses [62]. We have earlier shown that vaccinia virus (VV) evaded the NKT cell activity by inhibiting the CD1d-mediated antigen presentation through the alteration of the mitogen-activated protein kinases (MAPKs) (Figure 3) [63]. A VV infection inhibited the CD1d-mediated antigen presentation by activating the p38 MAPK. The inhibition of p38 MAPK signaling using a specific inhibitor SB203580 rescued the VV-induced inhibition of CD1d-mediated antigen presentation. The alteration of MAPKs activation changed the intracellular trafficking of CD1d through the ligand-loading endocytic compartments [63]. In another study, we demonstrated that JNK2 negatively regulated the antigen presentation by CD1d and also contributed to the IL-12 induced activation of iNKT cells [64].

The signal transducer and activator of transcription-3 (STAT-3) has been shown to activate NKT cells by promoting CD1d-mediated antigen presentation [65]. The STAT-3 signaling induces an antiviral immunity as the treatment with a specific STAT-3 inhibitor increased the viral load in VV-infected mice [66]. In an another study, we and colleagues showed that a vesicular stomatitis virus (VSV) inhibited the CD1d-mediated antigen presentation much faster as compared to that inhibited by VV. A matrix protein, M protein, of VSV played a key role in inhibiting CD1d-mediated antigen presentation [67]. Moreover, a chronic LCMV infection caused a long-term loss of NKT cells in mice [68,69]. Interestingly, the acute infection of mice with LCMV caused a reduction in CD1d cell surface expression on dendritic cells and macrophages [68]. Moreover, West Nile virus (WNV) interferes with the interaction of dendritic cells (DCs) with NKT cells and thus inhibits the secretion of proinflammatory cytokines [70]. Kovats et al. showed that WNV-infected human dendritic cells failed to fully activate NKT cells [70]. HSV-1 has been shown to inhibit CD1d-mediated antigen presentation by suppressing the recycling of CD1d on the cell surface [71]. Viral glycoprotein protein (gB) and viral serine-threonine kinase US3 are required to inhibit the CD1d antigen presentation and NKT cell activation.

Programmed death (PD)-1 is a member of the CD28 family of the costimulatory molecules and its interaction with its ligands PD-L1 and PD-L2 on APC sends the inhibitory signals to T cells [72]. The PD-1:PD-L interaction contributes to the induction of NKT cell anergy [73]. It has been shown that an increased expression of PD-1 on the T cells in HIV-1 infection induces T cell exhaustion and contributes to the progression of the disease [74]. Moll et al. demonstrated the elevated expression of PD-1 and functional impairment in CD1d-restricted NKT cells in the chronic infection of HIV-1 [56]. The coadministration of anti-PDL1 monoclonal antibody and α-GalCer modulated the NKT cell activity and inhibited HBV infection [44]. Recently, Zingaropoli et al. showed a reduction in NKT cells in the peripheral blood of COVID-19 patients [75].

## 5. Nanotechnology-Based Vaccine Delivery Platforms and the Development of an NKT Cell-Based Nanovaccine

The development of nanotechnology-based formulations has shown great promise for the development of new generation vaccines. The nanoparticle-based delivery systems not only improve the stability of the vaccine, but also increase the immunogenicity of the encapsulated antigens by delivering them to the intracellular locations of APCs. The shape, size and the surface properties of the delivery systems determine their efficacy as an immunoadjuvant. Various types of vaccine delivery systems, including gold and silver nanoparticles, liposomes and chitosan nanoparticles have the ability to induce the antigen-specific T cell responses and antibody responses [76]. The important vaccine nanocarriers include liposomes, inorganic nanoparticles, chitosan nanoparticles, PLGA nanoparticles and virus-like particles (VLPs) [77,78].

### 5.1. Inorganic Nanoparticles

Several inorganic nanoparticles, including gold, silver, silica and iron have been formulated as vaccine delivery systems [79]. Influenza virus M2 membrane protein immobilized on gold-nanoparticles induced protective immunity against influenza A subtypes [80]. Silica nanoparticles loaded with CV2-ORF2 proteins elicited antigen-specific cell- and antibody-mediated immune responses [81]. Carbon nanotube-conjugated peptides from the foot-and-mouth disease virus elicited a strong antigen-specific immune response [82].

### 5.2. Liposomes

Liposomes are the unilamellar or multilamellar lipid vesicles that are composed of biodegradable phospholipids, including phosphatidylserine, phosphatidylcholine and cholesterol. Liposomes deliver the antigens to the cytoplasmic compartments by fusing with the membrane of the APCs [78]. The modification of the liposomal surface with certain molecules increases the immunoadjuvant potential of the liposomes [83,84]. Liposomes composed of cationic lipids elicited a strong immune response against the hepatitis B surface antigen (HBsAg) [85]. In addition, liposomes have been shown to be very effective in inducing antigen-specific immune responses against IAV and RSV [86,87].

### 5.3. Polymeric Nanoparticles

The polymer-based vaccine carriers are composed of biodegradable polymers, including chitosan, polylactic acid, polyglutamic acid, and poly(lactic-glycolic acid) [88]. Among them, the chitosan-based nanoparticle vaccine carriers have been extensively studied due to their biodegradability and reduced toxicity [89]. Chitosan-bearing nanoparticles have been shown to improve the immunogenicity of the influenza virus vaccine [90]. The design of poly(lactic-co-glycolic acid) (PLGA) nanoparticles improved the antigen-specific immune responses [91] Moreover, PLGA nanoparticles increased the antigen-specific lymphocyte proliferation against H1N2 antigens [92].

**Figure 4:**
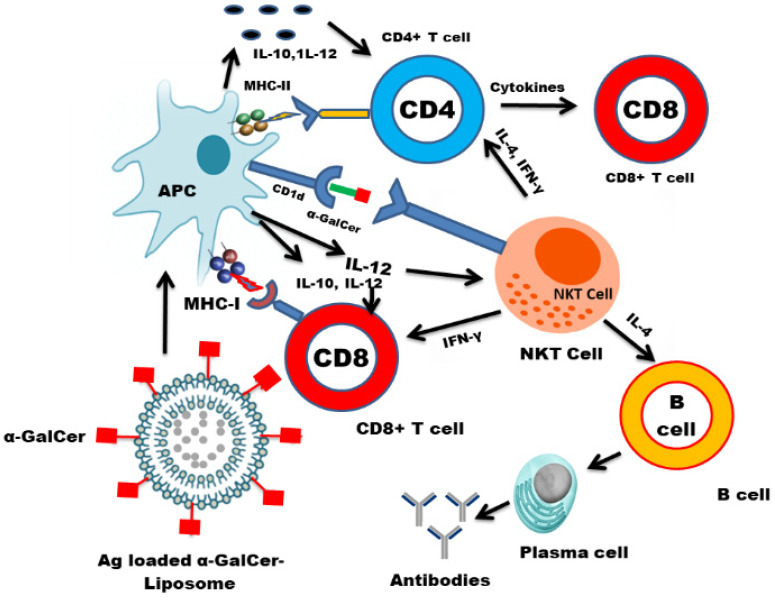
Liposome-incorporated α-GalCer and antigens are preferentially taken up and presented by dendritic cells. Liposomes deliver the encapsulated molecules to the cytoplasm and intracellular compartments of the APC for the processing of antigens. It results in the activation of CD8^+^ T cells, CD4^+^ T cells and NKT cells. NKT cells secrete both Th1 and Th2 cytokines that induce the proliferation of the cell-mediated and humoral immune responses.

A successful vaccine should be able to induce a strong CD8^+^ T cell immune response in order to protect against viral infections (Figure 4). Generally, the vaccines composed of the live attenuated pathogens can generate CD8^+^ T-cell-mediated immune responses [93,94]. The antiviral activity of the NKT cells is evident from the fact that NKT-cell-deficient mice showed a greater susceptibility to viruses [95,96]. Since NKT cells have a unique ability to stimulate the innate and adaptive immune responses, the NKT-cell ligand may prove to be an effective immunoadjuvant in the development of a successful vaccine.

**Figure 5:**
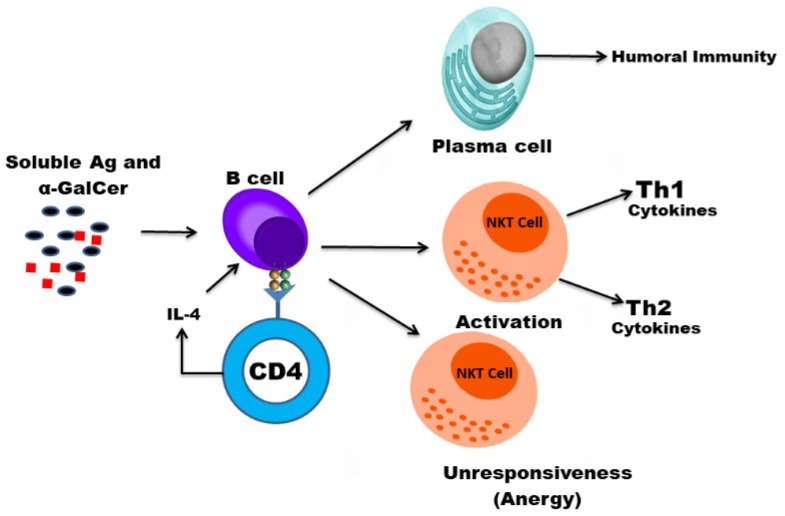
Soluble α-GalCer presented by B cells anergize NKT cells. In contrast to dendritic cells, α-GalCer-pulsed B cells express low levels of costimulatory molecules and anergize NKT cells.

The NKT cells show immune-stimulating activity through the maturation of the dendritic cells and boosting of antibody production [97,98]. Since they induce a rapid maturation of the dendritic cells (DCs) and B cells, their activation may elicit a strong antigen-specific immune responses. Various studies have shown the immunoadjuvant effect of α-GalCer in protection against influenza virus infections [99,100]. The use of a synthetic glycolipid ABX196, an NKT ligand, demonstrated an immunoadjuvant effect and stimulated an anti-HB antibody response in human volunteers [101]. Huang et al. reported that α-GalCer enhanced the immunogenicity of the DNA vaccine by increasing antigen-specific T-cell and antibody immune responses [102]. A major drawback with the use of α-GalCer is that its sequential systemic administration results in the NKT cell anergy that hampers the development of the NKT-based immunotherapeutics (Figure 5). Moreover, the presentation of α-GalCer by the nonprofessional APCs may induce NKT cell anergy. In order to exploit the NKT cell-mediated immune stimulation, it is important to codeliver the NKT cell ligand and antigens to the professional APCs such as dendritic cells. The co-administration of α-GalCer and soluble antigens enhanced the antigen-specific cell-mediated and humoral immunity [103]. However, the coadministration of α-GalCer and soluble antigens does not necessarily ensure that they are taken up by the same APC. In order to fully exploit the iNKT role to induce an effective CD8^+^ T-cell response, it is essential that α-GalCer and antigens are delivered to the same professional APC. This goal can be achieved by using the nanoparticles as delivery systems for antigens and NKT cell ligand. The liposome-mediated delivery of α-GalCer and tumor-associated antigens (TAA) has resulted in an increased antigen-specific CD8^+^ T-cell response [104]. Another study showed that the delivery of α-GalCer with ovalbumin (OVA) in poly(lactic-co-glycolic acid) (PLGA) nanoparticles induced a very strong antigen-specific CD8^+^ T-cell response [105].

We have earlier shown that liposomes can fuse with the membrane of APC and deliver the antigens to the cytoplasmic compartment for processing [74]. These antigens are presented by MHC class I molecules to induce the activation of CD8^+^ T cells. The activation of antigen-specific CTLs is critical in the development of an antiviral vaccine. Moreover, the liposome-encapsulated antigens are also taken by the APCs through phagocytosis where the antigens are presented by MHC class II molecules to CD4^+^ T cells resulting in the secretion of various cytokines. On the other hand, MHC class I-like CD1d molecules present glycolipid antigens to NKT cells. The iNKT cell-specific ligands recruit NKT cells, like CD4^+^ T cells, that play an important role in the modulation of the humoral and cytotoxic T-cell responses. The stimulation of NKT cells leads to the activation of downstream immune cells, including NK cells, DCs, macrophages, B cells, and conventional T cells. Many of these immune cells secrete immune-modulating cytokines, creating an entire activation cascade. 

In contrast to DCs, B cells express lower levels of costimulatory molecules and contribute to NKT cell anergy [106]. Interestingly, liposome-incorporated-α-GalCer is preferentially taken up by the dendritic cells and activates iNKT cells without inducing anergy [107]. Thapa et al. demonstrated that the soluble α-GalCer is presented by B cells, whereas the nanoparticle-incorporated α-GalCer is preferentially presented by DCs. Moreover, glycosphingolipids (GSLs), isolated from *S. paucimobilis*, specifically activated the iNKT cells in a CD1d-dependent manner [17]. Liposomes composed of GSLs elicited a strong antigen-specific immune response [108]. Moreover, they induced the activation of DCs and increased the production of IFN-γ by the splenocytes [108,109]. It suggested that the incorporation of iNKT-specific ligands in nanoparticles or liposomes may further potentiate the immunogenicity of encapsulated antigens by enhancing the generation of antigen-specific CD8 ^+^ T cells and CD4 ^+^ T cells that contribute to the development of an effective vaccine against viruses. 

## 6. Conclusions

In spite of constituting a very small population of T cells, NKT cells play a very critical role in protecting against viruses. They have both the direct and indirect antiviral effect through the secretion of important cytokines. Contrarily, viruses evade the functioning of the NKT cells by utilizing multiple immune evasion mechanisms. While the NKT-cell ligands have been extensively studied as a potential therapeutic agent in the treatment of cancer, their role, however, as immunoadjuvants has not been widely explored. Some studies showed that NKT-cell ligands have immunoadjuvant potential in eliciting the antigen-specific cell-mediated and humoral immune responses. Nevertheless, their frequent systemic administration results in NKT cell anergy. Nanoparticle-based vaccine formulations have shown their efficacy in the generation of CD4^+^ and CD8^+^ T cell responses, the latter is critical to control intracellular infections, particularly viral infections. The codelivery of NKT cell ligand and antigens by nanoparticles is suggested to be a very important strategy to prepare an antiviral vaccine because it will not only activate CD4^+^ and CD8^+^ T cells, but also stimulate iNKT cells that further contribute in the activation of the cell-mediated and humoral immune responses through the secretion of various cytokines. Moreover, iNKT cell ligand incorporated into nanoparticles can persistently activate iNKT cells without inducing anergy. Thus the codelivery of nanoparticle-incorporated iNKT cell ligand and antigens may be considered an important prophylactic approach to mount a strong antigen-specific antiviral immune response.

## Data Availability

Not applicable.
